# Cardiac angiosarcoma treated with 1.5 Tesla MR-guided adaptive stereotactic body radiotherapy – Case report and review of the literature

**DOI:** 10.1016/j.ijscr.2022.107521

**Published:** 2022-08-15

**Authors:** Asli Noyan, Guler Yavas, Esma Efe, Gungor Arslan, Cagdas Yavas, Cem Onal

**Affiliations:** a Baskent University, Faculty of Medicine, Ankara, Turkey; b Baskent University, Faculty of Medicine, Department of Radiation Oncology, Ankara, Turkey; c Baskent University Faculty of Medicine, Adana Dr. Turgut Noyan Research and Treatment Center, Department of Radiation Oncology, Adana, Turkey

**Keywords:** Cardiac tumor, Angiosarcoma, Radiotherapy, MR-guided radiotherapy, Stereotactic body radiotherapy

## Abstract

**Introduction:**

Cardiac angiosarcoma is a very rare disease. As a result of their nonspecific presentation symptoms, and the lack of consensus in treatment, caution should be taken in both diagnosis and treatment. The role of radiotherapy (RT) is debatable due to the continuous movement of the heart, which makes it difficult to safely deliver high radiation doses to the target volume.

**Presentation of case:**

The case of a 16-year-old boy with cardiac angiosarcoma that recurred one year after surgery and was treated with chemotherapy is presented. The patient received high field 1.5-Tesla (magnetic resonance) MR-Linac treatment in 5 fractions with a dosage of 25 Gy to the tumor bed and 30 Gy to the recurrent nodules using the simultaneous integrated boost technique. The patient tolerated the treatment well and had stable disease two months later.

**Discussion:**

MR-guided radiotherapy, particularly in the case of cardiac malignancies, allows for direct tumor visualization with high soft tissue image resolution capacity. Furthermore, modern RT techniques allow for the full therapeutic window to be used by achieving superior dose distributions, allowing for dose escalation strategies with tolerable toxicity rates.

**Conclusion:**

Magnetic resonance guided RT allows direct visualization of the target during treatment delivery, allowing for higher-dose administration with less damage to healthy tissue near the tumor. This treatment strategy is a viable option in selected patients with cardiac angiosarcoma.

## Highlights

• Cardiac angiosarcoma is a rare, difficult-to-diagnose and -treat disease.
• The role of radiotherapy is debatable due to the continuous movement of the heart.
• MRgRT allows direct target visualization during treatment delivery.
• This is the youngest case of cardiac sarcoma treated with adaptive MRgRT.
• Ultra-hypofractionated MRgRT is a viable treatment option for cardiac tumors.

## 1 Introduction

The prevalence of primary cardiac tumors is <0.1 %, and only 25 % of primary cardiac tumors are malignant, with angiosarcoma being the most common subtype [1], [2]. Although primary cardiac tumors were rarely diagnosed and reported at autopsies prior to the mid-1950s, the development of imaging technologies made diagnosis and treatment an exciting possibility [1]. However, cardiac tumors are still difficult to diagnose due to their nonspecific presentation symptoms, and there is no consensus regarding treatment due to disease rarity [3]. Although surgery is the preferred treatment for patients with cardiac angiosarcoma, only a small number of patients were surgical candidates. The majority of cases were lost due to local recurrence or distant metastasis, making the prognosis for these patients dismal [3], [4].

It was unsafe to administer higher radiation doses to the heart using conventional irradiation techniques due to limited image guidance. However, recent technological advancements in the field of radiation oncology may offer these patients new options. The introduction of magnetic resonance guided RT (MRgRT), in particular, allows for direct visualization of the target even during treatment delivery, allowing for higher-dose administration with less damage to healthy tissue near the tumor [5]. Although promising results demonstrated the feasibility of cardiac MRgRT also for non-oncological diseases in cases of refractory ventricular tachycardia and support the use of such advanced delivery techniques for cardiac irradiation, only a small number of patients with primary cardiac tumors were treated with MRgRT [6], [7]. We present the case with recurrent cardiac angiosarcoma one year after surgery and was treated with high field 1.5-Tesla (T) MR-linac using stereotactic MR-guided online adaptive radiation therapy (SMART) technique.

The work was reported in accordance with SCARE criteria and the revised SCARE guidelines for 2020 [8].

## 2 Case presentation

A 16-year-old boy complained of fatigue, anorexia, and abdominal distention that did not respond to medication. The patient applied to the hospital as an outpatient with these complaints. The patient has no past medical and surgical history. On physical examination, the patient had ascites and tachycardia. An echocardiogram revealed an intra-cardiac mass in the right atrium measuring 85 cm. The mass invading the superior vena cava was seen on computed tomography (CT), and a pericardial effusion measuring 24 mm at its thickest point was demonstrated. The tumor was surgically removed and measured 9.5 × 6.5 × 4.3 cm and weighed 126 g. Pathology revealed that the patient had angiosarcoma with malignant pericardial effusion. Tumor cells were diffusely positive for CD31, CD34, ERG, and FLI1, as well as focally positive for CD117. There was moderate atypia, >50 % necrosis, and 18 mitoses per high power field. The Ki-67 proliferation index was found to be as high as 50 % at some areas. The surgical margin was positive.

**Fig. 1:**
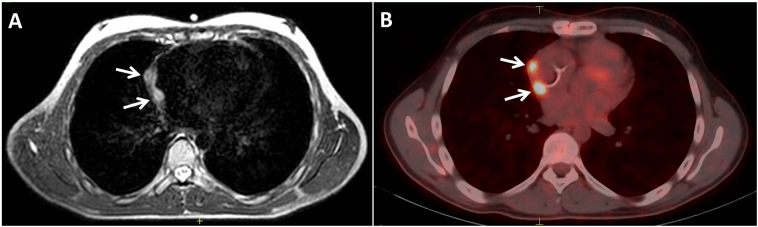
Recurrent nodular lesions (arrows) located at the right atrial wall demonstrated in (A) magnetic resonance imaging and (B) 18-flourodeoxyglucose positron emission tomography.

At 18-Fluorodeoxyglucose positron emission tomography (FDG-PET/CT), no distant metastases were found. The patient underwent chemotherapy consisting of iphospahmide and doxorubicin according to the The European Pediatric Soft tissue Sarcoma Study Group / Non Rhabdomyosarcoma Soft Tissue Sarcomas 2005 protocol from May 2021 to November 2021. The last control imaging done in December 2021 revealed no recurrence or residual lesion. Three months later, the pediatric oncology board decided on follow-up imaging. Two new nodular lesions with 10 mm and 35 mm size were discovered in the right atrium in the control MRI 3 months after chemotherapy (Fig. 1A). On 18-flourodeoxy-glucose positron emission tomography (FDG-PET/CT) imaging, two lesions with increased FDG uptake were observed at the right atrium wall, measuring 40 mm (SUV = 4) and 11 mm (SUV = 8.4) (Fig. 1B).

**Fig. 2:**
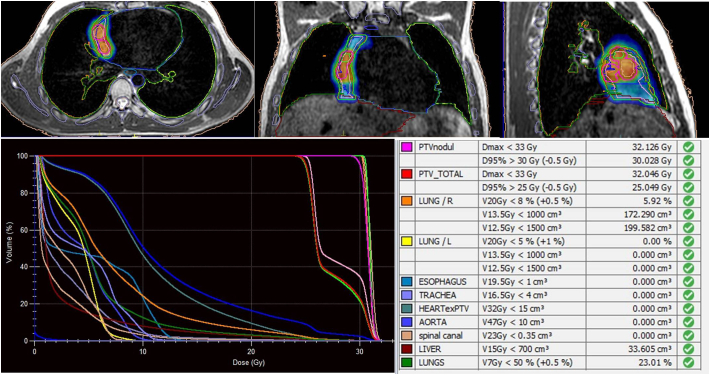
Dose distribution of stereotactic radiotherapy in 5 fractions using the simultaneous-integrated boost technique with 25 Gy to the tumor bed and 30 Gy to recurrent nodules. The dose volume histogram and dose constraints are used to determine the organs at risk doses.

**Fig. 3:**
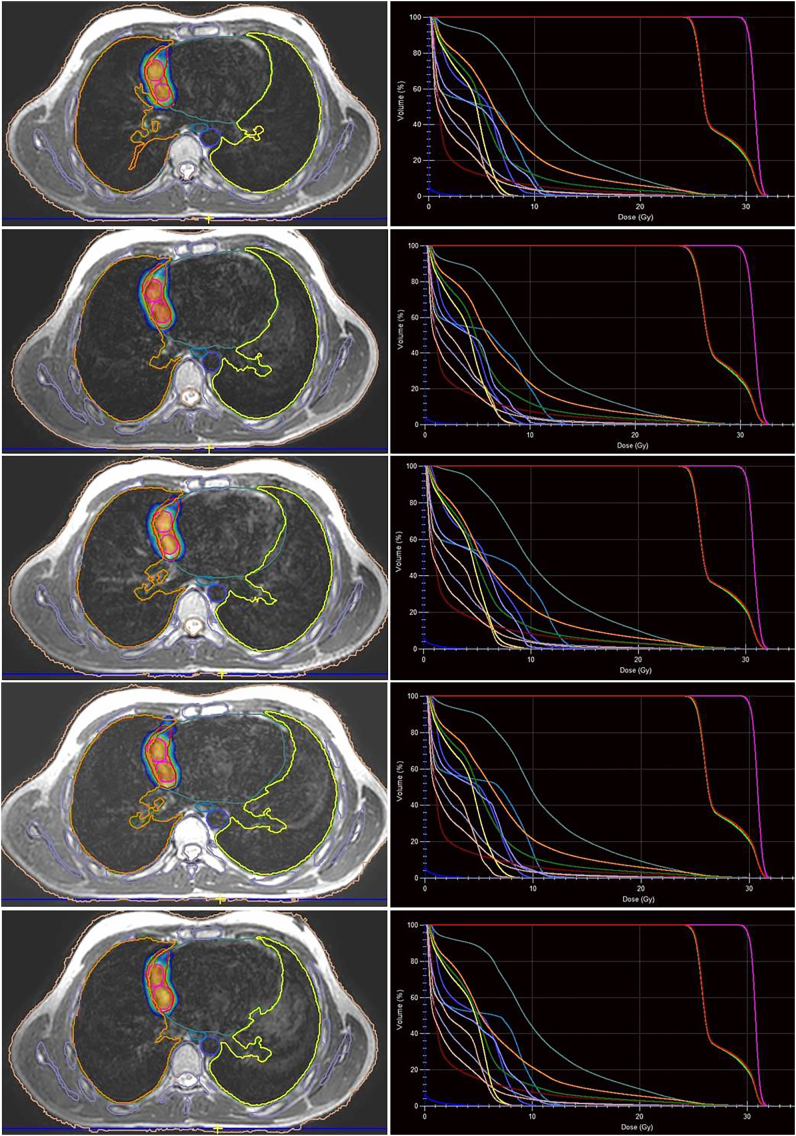
Dose distribution and dose-volume histograms for each fraction of the adapt-to-shape adaptive plan.

**Fig. 4:**
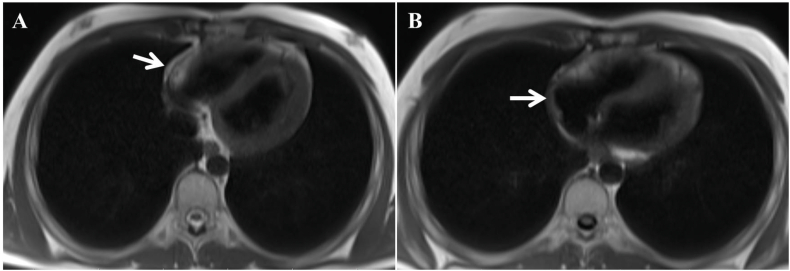
Magnetic resonance imaging at axial sections revealed (A) a stable nodular lesion (arrow), the disappearance of another nodular lesion, and (B) the absence of a new lesion at the tumor bed (arrow).

The patient was referred to radiation oncology department for treatment with MRgRT and he was treated with was treated with 1.5 T MR-Linac (Unity® MR Linac System, Elekta AB, Stockholm, Sweden) using simultaneous integrated boost (SIB) technique in 5 fractions to a dosage of 25 Gy to tumor bed, and 30 Gy to the recurrent nodules. The planning target volume (PTV) for 25 Gy was created by expanding the clinical target volume (CTV) by 3 mm in all directions and the PTV of 30 Gy was created by the 3 mm expansion of tumor nodules (Fig. 2). The total doses of 30 Gy to recurrent nodules and 25 Gy to tumor bed was delivered in 5 fractions every other day using an adapt-to-shape strategy (Fig. 3). To prevent arrhythmia, the patient continued to take propranolol throughout SBRT. The patient experienced no radiation-related acute toxicity during or after the MRgRT. One month after completion of SBRT, an MRI demonstrated a stable nodular lesion, the disappearance of a second nodular lesion, and the absence of a new lesion at the tumor bed (Fig. 4).The patient has a stable disease two months after finishing treatment and has not received any further treatment.

## 3 Discussion

Cardiac angiosarcoma is a rare type of soft tissue sarcoma with a poor prognosis due to its aggressive nature, high rates of local recurrence, and systemic metastases [9]. Several studies have shown that localized surgical excision produces the best long-term survival outcomes [10], [11], [12]. However, depending on the location of tumor or the presence of distant metastases at diagnosis, surgery may not be an option for some patients. In such cases, combined modality therapies combining RT and chemotherapy have emerged as a viable alternative [13].

Radiotherapy is challenging for cardiac tumors because the heartbeat and respiration make it difficult to focus the beam without harming the surrounding healthy tissue. The implementation of image-guided radiotherapy (IGRT) and stereotactic radiotherapy (SBRT) permits more accurate targeting of mobile tissue during treatment [14]. Additionally, the MR-Linac system allows for daily plan adjustments to account for position changes, and real-time MRgRT permits the acquisition of high-quality MR images prior to and throughout the treatment [15]. Particularly in the case of cardiac malignancies, MRgRT permits direct tumor visualization with high soft tissue image resolution capactiy. Furthermore, modern RT techniques enable the utilization of the entire therapeutic window by achieving superior dose distributions, thereby permitting dose escalation strategies with tolerable toxicity rates. SBRT, a well-established method for the ablative treatment, can deliver large doses of radiation in a small number of fractions for RT treatments within sensitive surrounding organs such as the heart. Numerous case reports and small case series detailing their treatment of primary heart sarcomas have been published [3], [5], [6], [13], [16] (Table 1). However, only a small number of cases utilized ultrahypofractionated SBRT [6], [7], and none utilized the simultaneous integrated boost technique to administer higher doses to gross lesions.

**Table 1:** Published cases involving patients treated MR-guided radiotherapy for cardiac angiosaroma.

Author-year	Age (years)	RT technique	RT dose	Toxicity	Outcome
Corradini 2021 [7]	49–80	0.35 T MR Linac	38.9 Gy (mean GTV dose) in 5 fractions	Fatigue, dyspnea, chest pain	Alive at follow up, one patient had lymph node and liver metastases
Pomp 2021 [6]	58	1.5 T MR Linac	60 Gy in 12 fractions	NA	Alive at follow up 6 months later with stable disease
Current case	16	1.5 T MR Linac	25–30 Gy in 5 fractions	No acute toxicity	Stable disease at 2 month follow up

Krishnan et al. [13] present a case report of a 46-year-old female patient who received 60 Gy in 30 fractions with weekly paclitaxel 80 mg/m^2^. They do, however, report complications such as severe esophagitis that necessitated nasogastric feeding tube and radiation pneumonitis. Clinical improvement occurred over time, and the tumor shrank in size, allowing the patient to undergo surgical resection. Five months after surgery, the patient was still asymptomatic. Fields et al. [16] presented a patient with cardiac angiosarcoma who received emergency high-dose single-fraction RT of 8 Gy, followed by 50 Gy in 25 fractions and paclitaxel weekly. The patient was free of acute toxicity and disease progression one month after treatment and had responded well to treatment. Corradini et al. [5] reported four patients treated with a 0.35 T hybrid MR Linac system (MRIdian, ViewRay Inc., Mountain View, CA) and a mean GTV dose of 38.9 Gy (range: 30.1–41.1 Gy) delivered in 5 fractions. Pomp et al. [6] describe a patient with recurrent cardiac sarcoma after surgery who was treated in the Netherlands with a 7-MV linear accelerator and an integrated high energy 1.5-T MR scanner. Over the course of four weeks, the tumor was irradiated with a high dose of 60 Gy in 12 fractions. Six months after finishing treatment, the patient was stable. Our patient received ultra-hypofractionated SBRT using 1.5-T MRgRT using SIB technique with 25 Gy to tumor bed in 5 fractions and 30 Gy to recurrent nodules, and the patient tolerated the treatment well, with no toxicity during and two months after treatment completion. Our case differs from those of the past due to the use of 1.5-T MRgRT with SIB technique and a younger age.

## 4 Conclusion

Due to technical difficulties in delivering high radiation doses safely, RT is rarely used in highly selected patient populations to treat cardiac angiosarcoma. With the implementation of high field 1.5-T MR with RT devices, however, it is simple to identify tumors in soft tissues and to track organ motion throughout the treatment session. This innovative technique permits the delivery of hypofractionated RT with tight safety margins in order to reduce radiation doses to normal tissues in the nearby area. In certain cases, ultra-hypofractionated MRgRT is a viable treatment option for cardiac tumors. This is, to the best of our knowledge, the youngest case of primary cardiac sarcomas treated with ultra-hypofractionated SBRT delivered by online adaptive MRgRT with a 1.5-T MR linac. However, longer follow-up and additional research are necessary to determine the effect of this new method on the outcome of this extremely rare disease.

## Provenance and peer review

Not commissioned, externally peer-reviewed.

## Consent

Written informed consent was obtained from the patient for publication of this case report and accompanying images. A copy of the written consent is available for review by the Editor-in-Chief of this journal on request.

## Guarantor

Cem Onal, MD.

## Ethical approval

This case report does not require any ethical approval.

## Funding

None.

## Registration of research studies

Not applicable.

## CRediT authorship contribution statement

Asli Noyan MD - Study concept, writing the paper, final draft.

Guler Yavas, MD - Study concept, writing the paper, final draft.

Esma Efe, MSci - Data collection, review of literature.

Gungor Arslan, MSci - Data collection, review of literature, final draft.

Cagdas Yavas, MD - Data curation, resources, investigation, visualization.

Cem Onal, MD - Conceptualization, formal analysis, investigation, writing - review & editing, supervision.

## Declaration of competing interest

None.
